# Life-Threatening Bleeding From Acquired FXI Inhibitors in a Patient With Colorectal Adenocarcinoma

**DOI:** 10.1155/crh/3821648

**Published:** 2025-06-27

**Authors:** Marina Dragičević Jojkić, Amir El Farra, Nebojša Rajić, Ivana Urošević, Aleksandar Savić

**Affiliations:** ^1^Department of Internal Medicine, Faculty of Medicine, University of Novi Sad, Novi Sad, Vojvodina, Serbia; ^2^Department of Hematology, Clinical Center of Vojvodina, Novi Sad, Vojvodina, Serbia; ^3^Department of Internal Medicine, General Hospital Djordje Jovanovic, Zrenjanin, Vojvodina, Serbia

## Abstract

Acquired inhibitors of coagulation factor XI (FXI) are a rare cause of bleeding disorders, typically associated with autoimmune diseases or malignancies. Although uncommon, these inhibitors can lead to severe bleeding, which can be difficult to manage. A limited number of cases have been reported where acquired FXI inhibitors are associated with malignancy. This case report presented a rare occurrence of acquired coagulation FXI inhibitors in a 60-year-old male with sigmoid colon adenocarcinoma. The patient experienced severe postpolypectomy gastrointestinal bleeding and was diagnosed with FXI inhibitors after laboratory tests revealed prolonged activated partial thromboplastin time (aPTT) and reduced activities of factors IX, XI, and XII. The patient underwent surgery, and life-threatening hemorrhagic shock developed. He was reoperated, and treatment with recombinant factor VIIa (rFVIIa), tranexamic acid, and oral corticosteroids was initiated. The therapy successfully controlled the bleeding and resolved the inhibitor. This case highlights the risk of severe bleeding in patients with acquired FXI inhibitors and emphasizes the importance of early diagnosis and personalized treatment. Regular monitoring is essential due to the risk of relapse, particularly in cases associated with malignancy.

## 1. Introduction

Coagulation factor autoantibodies are pathologically acquired circulating immunoglobulins which inactivate specific coagulation factors or inhibit the cascade of the coagulation pathway by complex mechanisms [1]. The development of acquired inhibitors of coagulation factor XI (FXI) in patients without congenital deficiency is an extremely rare phenomenon and has been reported mostly in patients with autoimmune diseases or in association with malignancies [2, 3]. The risk of bleeding events in patients with FXI deficiency is relatively low and spontaneous bleeding is not common. In these patients, hemorrhage is usually provoked, exacerbated by trauma, or surgical procedures. Even though bleeding in FXI deficiency patients is not severe, invasive procedures can cause serious and unpredictable hemorrhage [3, 4].

## 2. Case Presentation

**Figure 1:**
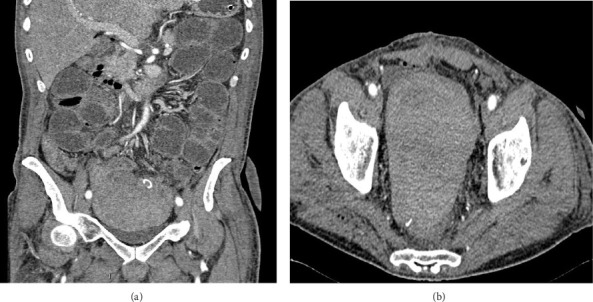
Computed tomography of the abdomen showing retroperitoneal hematoma (a) and axial view (b).

A 60-year-old man was admitted to the emergency department due to rectorrahgia after a polypectomy which was performed 10 days prior. He had a history of atrial fibrillation, which was, at that moment, treated with low molecular weight heparin. He had stopped taking oral anticoagulants for the past month. He did not have a history of hemorrhagic diathesis or a family history of bleeding disorders. On admission, the laboratory findings showed mild anemia, hemoglobin of 10.9 g/dL, platelet count of 297 × 10^9^/L, and total leukocytes of 7 × 10^9^/L. The blood levels of glucose, creatinine, total bilirubin, alanine aminotransferase, glutamic oxaloacetic transaminase, γ-glutamyltransferase, alkaline phosphatase, amylase, total proteins, C reactive protein, and electrolytes were normal. The coagulation tests revealed prolonged activated partial thromboplastin time (aPTT) 39.5 s, R 1.61 (normal range: 25.0–33.5 s; *R* < 1.3) with normal prothrombin time (PT), thrombin time, and fibrinogen values. The colonoscopy showed bleeding in the descending colon and a large bleeding coagulum in the sigmoid colon. The histopathological diagnosis of the previously removed colorectal polyp was adenocarcinoma. No affected lymph nodes or distant metastasis were detected. The clinical staging of the tumor was T2N0M0. Due to the diagnosis of adenocarcinoma and profuse bleeding which could not be stopped during colonoscopy, the patient had to undergo surgery. Resection of the sigmoid colon was performed with the creation of a terminal colonostomy. The patient had received two doses of fresh frozen plasma and the control aPTT value was 32.5 s, R 1.29, and the postoperative course was normal. On the 14th postoperative day, serosanguineous drainage appeared and the patient started to complain of abdominal pain. A retroperitoneal hematoma (10 × 10 cm) was verified by computed tomography so surgical evacuation of the hematoma was perfomed (Figures 1(a) and 1(b)).

Preoperative laboratory investigation revealed a hemoglobin level of 11.9 g/dL, platelet count of 685 × 10^9^/L, leucocytes of 20.4 × 10^9^/L, and aPTT 39.7 s, R 1.62. The patient received two doses of fresh frozen plasma and one dose of cryoprecipitate. A few hours later, the patient's condition deteriorated, he had a large bloody stool and blood appeared in the abdominal drains and consequently a hemorrhagic shock developed (a drop in hemoglobin level to 38 g/L). Due to the patient's severe hemodynamic instability, an urgent reoperation was performed to locate the bleeding site. About 2900 mL of blood and coagulums were removed from the peritoneal cavity. The value of aPTT was R 1.75, PT R 1.43, and fibrinogen level 1.60 g/L. After surgery, the patient was admitted to the Intensive Care Unit as he required respiratory and vasoactive support while resuming supstitution with blood and blood derivatives. Additional laboratory analysis with rotational thromboelastrometry (ROTEM) was performed. Deficiency of factors IX (54%), XI (31%), and XII (31%) were detected. To investigate the prolonged aPTT, mixing studies were conducted. After 2 hours of incubation at 37°C, the result of 1:1 mixing study (patient plasma mixed with normal pooled plasma) showed no correction of the aPTT, consistent with the presence of an inhibitor. Bethesda assay analysis established the presence of an acquired FXI inhibitor, with an inhibitor titer quantified at 1.25 BU/mL (Table 1). Lupus anticoagulant was ruled out. Administration of recombinant factor VIIa (rFVIIa; NovoSeven, Novo Nordisk—90 μg/kg) was started along with tranexamic acid (1 g/8 h) and prednisolone (1 mg/kg). On the next day, the patient's condition improved and the bleeding intensity decreased, which stopped on the second postoperative day.

The dosages of rFVIIa and intervals of administration were adjusted according to the clinical and laboratory signs of bleeding (90 μg/kg every 2-3 h with the extension of the interval when bleeding control was achieved and stopped after three days). Antifibrinolytic and corticosteroid therapy was continued for the next 15 days. The patient's condition was stabilized and he was discharged on the 20th postoperative day, without any signs of bleeding and with the clotting tests within reference range. The activity of the coagulation factors was normal, while FXI inhibitors were no longer detectable. The patient was discharged from hospital using prednisone 40 mg/d for next 20 days when the therapy stopped. On discharge, the patient was advised to take Prednison 40 mg daily, with dose tapering over the next 20 days, after which the treatment was discontinued. The patient remained in remission, with no signs of relapse or postoperative and bleeding complications.

## 3. Discussion

Development of coagulation factor autoantibodies is a very rare coagulopathy, usually present in the elderly, with an overall incidence of 1.5 per million per year. Inhibitors are most commonly directed against factor VIII (75%–90% of all cases of acquired inhibitors) and von Willebrand factor (VWf), while inhibitors against other coagulation factors, including FXI, are only occasionally reported [5–12]. The incidence of acquired inhibitors to FXI is still unknown. The clinical presentation due to FXI inhibitors differs; most bleeding reports are mild but sometimes can be life-threatening bleeding [8–10]. Usually, bleeding phenotype is often parallel to the course of the underlying disease, but, approximately, half of the cases are idiopathic, with unidentified course [9, 13]. Spontaneous hemorrhagic episodes are not features of FXI deficiency. Bleeding may be seen in the oral and nasal cavity, pharynx, and genitourinary tract, which are areas of increased fibrinolytic activity. Common bleeding manifestations include epistaxis, hematoma, hematuria, menorrhagia, postpartal bleeding, and postoperative hemorrhage. Contrary to inherited hemophilia, hemarthroses is rare in acquired hemophilia [14–17].

In this case report, we described a case of acquired inhibitor to FXI with severe postpolypectomy bleeding and colon adenocarcinoma. We even detected inhibitors to FXI at low titer (1.25 BU) and a nonsevere reduction of FXI coagulant activity (FXI 31%) when the patient developed life-threatening bleeding and hemorrhagic shock. Based on this case, we can conclude that there seems to be weak correlation between the plasma FXI inhibitor concentration, factor activity and bleeding intensity. Other authors also described patients with severe life-threatening hemorrhage and low titer inhibitor to FXI [8, 11]. Wheeler asserts that the correlation between factor level activity and clinical symptoms is the weakest for FXI [18]. But, on the other hand, there is the fact that bleeding most likely occurs in individuals with FXI levels lower than 20%, which is a threshold for severe FXI deficiency [19]. Our patient was also diagnosed with FIX and FXII deficiencies, which is not surprising due to patients with FXI inhibitors often exhibiting low activity of FXII, precallicrein, kininogen, or even factors VIII and IX. Autoantibodies appeared to be directed against FXI but also other clotting factors participating in the intrinsic pathway. The intrinsic pathway is clinically measured as the aPTT, whose prolongation with the presence of soft tissue hematoma and/or bleeding is the main laboratory hallmark of acquired inhibitors of FXI [20, 21].

There are two principal goals in the treatment of acquired inhibitors of coagulation factors: controlling the bleeding as a short-term goal and inhibitor eradication as a long-term goal. Other objectives include the prevention of bleeding episodes and the treatment of the underlying disease [22, 23]. There are currently no recommended guidelines or standardized protocols available regarding the management of acquired FXI inhibitors. The 2013 UKHCDO guidelines recommend a case-by-case approach due to the fact that some patients with acquired factor inhibitors (other than FVIII) who are asymptomatic may not require treatment due to some inhibitors being transient and not causing clinical bleeding, despite significant laboratory abnormalities [24]. Whereas bleeding symptoms are a variable even at relatively low levels of FXI, the mainstream treatment decision is based on the clinical presentation, not to the plasma level of FXI [9]. It is highly recommended that patients with acquired clotting factor inhibitors and bleeding, which can be unpredictable and unexpected, need to be urgently admitted and managed in specialized centers with relevant experience in treating such patients [6]. Treatment regiments are varied, ranging from the no therapy and “watch and wait” approach, to high doses of corticosteroids, immunoglobulins, immunosuppressants, plasmapheresis, or massive doses of a coagulant factor that can neutralize the inhibitor, especially if they are at a low titer (< 5 BU) [6, 20]. Administration of bypass hemostatic agents, recombinant activated factor VII (rFVIIa) or FEIBA, and activated prothrombin complex concentrate may be considered as the first-line approach in treating life-threatening bleeding episodes [1, 24–26]. Considering that our patient developed severe life-threatening hemorrhagic shock, we administered recombinant factor VIIa which successfully stopped the bleeding. In addition, we started reducing the levels of autoantibodies with corticosteroid therapy, which provided a rapid response with the disappearance of the inhibitor on the 10^th^ day of therapy. An oral antifibrinolytic agent—tranexamic acid was added as an extra hemostatic factor. Supplementation with antifibrinolytic therapy should be used cautiously because of the potential for thrombotic events, especially in older patients and patients with comorbidities [6, 18].

In the case of underlying cancer, which probably triggered the development of the inhibitor in our patient, effective treatment either by surgery, by chemotherapy, or radiotherapy should be proposed and prioritized since the cancer remission usually leads to the disappearance of the inhibitor. By contrast, if the cancer is not removed and resolved, the inhibitor's eradication would not be successful. Sometimes, acquired clotting factor inhibitors may represent initial presentation, relapses of malignancy, or can be correlated with the aggressiveness of cancer cells. Previous studies reported that patients in whom the disappearance of the inhibitor was not attained by therapy had, usually, untreatable malignancy [6, 9, 27–29]. The use of rFVIIa in combination with tranexamic acid was effective in stopping life-threatening bleeding in our patient. The FXI inhibitor disappeared after the underlying disease went into complete remission. The exact mechanism by which cancer cells induce autoantibodies to clotting factors is not fully understood yet [30]. Only several cancers have been reported, mainly hematological malignancies, to be associated with autoantibodies to FXI [10, 12, 20, 31, 32]. Only Kyriakou acquired inhibitors of FXI in patients with gastrointestinal adenocarcinoma. The patient successfully responded within 3 weeks after surgical cancer resection [31]. Patients in other described cases did not bleed or had relatively mild bleeding symptoms, which did not require specific therapy, or they responded well to corticosteroid therapy.

## 4. Conclusion

Acquired inhibitors of coagulation FXI are a rare clinical entity, sometimes presenting a medical emergency and should be suspected in the presence of unexplained bleeding and a prolonged aPTT. Making the right diagnosis can be complicated, so it is not surprising that delays in diagnosis and treatment are common. Early and urgent diagnosis, together with prompt treatment, is crucial to improving the outcomes for these patients. In this case report, we describe a patient with postpolipectomy excessive bleeding and acquired inhibitors against FXI secondary to adenocarcinoma of the sigmoid colon. Despite the fact that bleeding phenotype in the presence of these inhibitors is mostly mild, sometimes bleeding can be severe and life-threatening, as we described above. Fortunately, in our case, the bleeding stopped, and eradication of the inhibitor was achieved with a combination of corticosteroid therapy and rFVIIa. Adequate treatment should be individualized according to the severity of the bleeding and the patient's comorbidities. Although our patient did not develop a relapse of the inhibitor within the following years, relapses are common, which makes regular biological and clinical monitoring of the inhibitor necessary, as well as follow-ups with the patient.

## Figures and Tables

**Table 1:** Laboratory values.

	1 month before admission	At admission	On the day of operation	On the 2^nd^postoperative day	On the 14^th^postoperative day (10 h before reoperation)	On the 14^th^postoperative day (2 h before reoperation)	At discharge	Reference values
RBC (10^12^/L)	4.0	2.8	3.1	3.0	3.8	1.4	3.2	4.00–5.50
Hgb (g/dL)	12.2	10.9	10.3	9.8	11.9	3.8	10.5	12.0–15.0
Hct	0.36	0.34	0.31	0.29	0.34	0.12	0.34	0.40–0.50
WBC (10^9^/L)	9.7	7.03	14.6	15.6	20.4	15.22	7.43	4.0–10.0
Plt (10^9^/L)	229	297	661	672	685	164	354	150–400
aPTT (R)	1.29	1.61	1.56	1.29	1.62	1.75	1.27	0.70–1.30
PT (R)	1.18	1.09	1.28	1.27	1.31	1.43	1.23	0.70–1.30
TT (R)	1.07	1.08	1.2	1.07	1.10	1.08	1.1	0.70–1.20
Fibrinogen (g/L)	3.3	3.2	2.9	3.1	3.0	1.6	2.9	1.8–3.5
F VIII						80%	122%	60…150%
F IX						54%	82%	60…150%
F XI						31%	75%	60…150%
F XII						31%	60%	60…150%
Inhibitor to F XI (BU/mL)						1.25	—	

## Data Availability

The data that support the findings of this study are available from the corresponding author upon reasonable request.
